# Mesothelioma: Do asbestos and carbon nanotubes pose the same health risk?

**DOI:** 10.1186/1743-8977-6-16

**Published:** 2009-06-12

**Authors:** Marie-Claude F Jaurand, Annie Renier, Julien Daubriac

**Affiliations:** 1 INSERM, U674, Fondation Jean Dausset – CEPH, Paris, F-75010, France; 2 Université Paris 7, Paris, F-75013, France

## Abstract

Carbon nanotubes (CNTs), the product of new technology, may be used in a wide range of applications. Because they present similarities to asbestos fibres in terms of their shape and size, it is legitimate to raise the question of their safety for human health. Recent animal and cellular studies suggest that CNTs elicit tissue and cell responses similar to those observed with asbestos fibres, which increases concern about the adverse biological effects of CNTs. While asbestos fibres' mechanisms of action are not fully understood, sufficient results are available to develop hypotheses about the significant factors underlying their damaging effects. This review will summarize the current state of knowledge about the biological effects of CNTs and will discuss to what extent they present similarities to those of asbestos fibres. Finally, the characteristics of asbestos known to be associated with toxicity will be analyzed to address the possible impact of CNTs.

## Introduction

Carbon nanotubes (CNTs) have unique chemical and physical characteristics as a result of their nanostructure. CNTs may be used in a wide range of applications, in fields as diverse as electronics and medicine [1,2]. Due to their widespread use, it is important to determine the safety of CNTs for the protection of ecological systems and human health. Research to investigate the biological effects of CNTs is advancing today in order to foresee and prevent their potentially harmful effects. CNTs have fibre-like characteristics in terms of their elongated shape, dimensions and aspect ratio. As particles with at least one dimension of less than 100 nm, they correspond to High Aspect Ratio Nanoparticles (HARN) [3]. In light of the health impact of mineral fibres, especially the fibrogenic and carcinogenic potency of asbestos fibres, and the health and socio-economical tragedies caused by unregulated asbestos utilization, the increasing development and uses of CNTs have triggered concern about their potential toxicity [4-8].

In recent years, several publications have reported the effects of CNTs. Most studies have concerned animal and cell responses, focusing primarily on respiratory diseases, especially the inflammatory effects in the lung. However, while inhalation is one important probable route of contamination, it must be kept in mind that there are other relevant routes of exposure. A severe primary cancer, malignant mesothelioma (MM), has been closely linked to asbestos exposure [9,10]. Epidemiological and animal studies have shown that asbestos fibres are not the only fibres to be associated with a risk of MM development. Epidemiological studies have demonstrated a higher incidence of MM in populations exposed to asbestiform and non-asbestos fibres [11-14]. Some manmade vitreous fibres have caused MM in animal experiments [15]. The question of whether CNTs might potentially be linked to MM development justifies further research in this area. Moreover, on the basis of the literature, CNTs have already shown effects in animals and in cell systems that are similar to those observed with asbestos fibres [1,2,5,7]. Two recent studies showed the occurrence of MM in genetically-modified cancer-sensitized mice and in conventional Fischer 344 rats exposed to CNTs by intraperitoneal or intrascrotal administration respectively [16,17]. These initial results underline the urgent need for information to further our knowledge about CNTs' potential to cause MM.

MM is a primary tumour of the serosas caused by the neoplastic transformation of mesothelial cells. In populations exposed to asbestos fibres, MM mainly occurs in the pleura, and to a lesser extent in the peritoneum and pericardium. MM is considered to be highly specific to asbestos exposure, and is found in from 60% to over 80% of cases [18-23]. In France, the calculated risk of MM attributable to occupational asbestos exposure was estimated at 83.2% (95% CI 76.8 to 89.6) in men, and 38.4% (95% CI 26.8 to 50.0) in women [24]. Many studies carried out to investigate pleural and mesothelial cell response to asbestos fibres have made it possible to reach sound hypotheses about the mechanism of action of asbestos fibres in neoplastic mesothelial cell transformation.

The aim of the present review is to explore whether our knowledge of the mechanism of action of asbestos fibres could offer a useful paradigm to provide a warning or predict the risk of CNTs, to interpret data on animal and cellular responses, and to evaluate their potential health effects. For the purposes of our discussion, we consider three points: (i) the fate of asbestos fibres following exposure; (ii) their effects on mesothelial cells and the biological mechanism associated with the cell response; (iii) the nature of the fibre parameters involved in the harmful effects, and their similarities with CNT characteristics. We begin with a summary of current knowledge on the toxicology of CNTs, then look at asbestos fibres' mechanisms of action, focusing on carcinogenic effects at the pleural level. Finally, we address the similarities between asbestos and CNTs.

## Toxicology of CNTs

### Context of toxicological studies on CNT

Various kinds of CNTS have been the focus of toxicological studies. CNTs are heterogeneous in terms of their structure, impurities and physico-chemical properties. Both single-walled (SWCNTs) and multi-walled (MWCNTs) CNTs have been examined in toxicological studies, including commercial and laboratory-made CNTs, whether purified or used as produced. The effects of CNTs have been investigated following *in vivo *exposure of rodents, and on several types of cells in culture. Most studies concerned pulmonary toxicity [1,2,5]. Animal experiments mainly focused on inflammatory responses after exposure by intratracheal instillation or aspiration, or intraperitoneal injection. *In vitro *cell systems with several types of mammalian cells have been used to study inflammatory responses and genotoxicity. A few *in vivo *and *in vitro *studies were related to dermal toxicity, and some *in vitro *studies focused on neurons [2]. Toxicity test systems on procaryotes were also used to assess genotoxicity. Here our focus will be on respiratory effects.

### Biological effects of CNTs

#### Translocation

Biodistribution of CNTs after deposition in the lung or *via *other routes has been poorly investigated. A translocation of SWCNTs in various organs has been reported by several authors [25-29]. In a recent study, MWCNTs deposited by intratracheal instillation in rats revealed clearance due to macrophage uptake and the lymphatic system without evidence of crossing the pulmonary barrier, six months after instillation [29]. It can be noted that macrophage and lymphatic clearance was also demonstrated following administration or exposure to asbestos fibres [30-33]. Erdely *et al*. [30] suggest that the release of soluble inflammatory factors could circulate to the vascular blood compartment after lung deposition of CNTs. The release of circulating factors must be taken into consideration to account for fibre effects. While asbestos fibres have been detected in the pleura, soluble molecules could also account for the pleural response [34], and genotoxicity may be due to clastogenic factors [35,36]. Additional studies are needed to determine the pharmacokinetics of CNTs. Regarding the numerous varieties of CNTs associated with a broad scale of physical and physico-chemical properties, fundamental studies will be necessary to establish the parameters leading the translocation process.

#### Biological effects on mesothelial cells

##### *In vivo *effects on mesothelial cells

Six recently-published studies concerned CNTs' effects on mesothelial cells. Three reported findings from animal experiments and three from cell system studies. One animal experiment concerned the mesothelial cell inflammatory response and pathological changes after intra-peritoneal injection [37]. The authors exposed C57Bl/6 mice to four samples of MWCNTs of different sizes and aggregation states. There was one sample of "short" MWCNTs (from NanoLab, Inc; mean diameter: 14.8 ± 0.5 nm; mean length: 1–5 μm); two samples of "long" MWCNTs (Long1, from Mitsui & Co.; mean diameter: 84.9 ± 1.9 nm; mean length: 40–50 μm [24% > 15 μm of length]; Long2 from Univ. Manchester; mean diameter: 165 ± 4.7 nm; mean length: 20–100 μm [84% > 15 μm of length]); and one sample of more tangled MWCNTs (from NanoLab, Inc.; mean diameter: 10.4 ± 0.3 nm; mean length: 5–20 μm), as well as carbon black. At the same time, two samples of amosite fibres were tested; these were short fibres (4.5% > 15 μm of length) and long fibres (50.4% > 15 μm of length) known to be differently pathogenic in rodents. In prior experiments, inhalation and intraperitoneal exposure in rats to long amosite fibres revealed greater pathogenicity than short fibres in terms of fibrosis and cancer [38,39]. In the study reported by Poland *et al*. [37], inflammation was assessed after injection of 50 μg of MWCNTs/mouse, after 24 h and seven days. The end points were quantification of inflammation in peritoneal lavage and histology of diaphragm. Only long samples of MWCNTs and of amosite produced inflammation and granulomas. Histological analyses revealed the occurrence of "frustrated phagocytosis" by macrophages. These results thus demonstrated some similarities between the responses to the long forms of amosite and MWCNTs. Several of the effects of asbestos were also found with CNTs. There were higher inflammatory responses with samples of long fibres. Only the samples that contained long fibres caused granulomas and "frustrated phagocytosis".

A long-term study was performed by Takagi *et al*. [17] who inoculated MWCNTs (MWCNTs-7 from Mitsui; diameter: 100 nm; length: 27% > 5 μm) in the peritoneal cavity of C57Bl/6 p53^+/- ^mice. Because these mice have a mutation in one allele of the *Trp53 *gene, they are prone to develop cancer. Crocidolite fibres were inoculated as positive control. Mesotheliomas were found after exposure to both MWCNTs and crocidolite. This study has been discussed on several points, including concern about the type of mice, inappropriate exposure methods, high exposure dose, underestimation of the number of particles of MWCNTs and poorly-illustrated histology [40,41]. Details can be found in the different papers but some of the authors' replies can be summarized here. It is recognized that *Trp53*^+/- ^mice are more prone to develop cancers, and that the response using high doses by the intraperitoneal route of exposure provides different information regarding hazard potency. However, spontaneous excess of mesotheliomas has not been reported in this type of mice, and the injection method is applicable to the hazard approach for mesothelial cells in the absence of human data. Concerning the dose, the authors mentioned that other experiments using lower doses are in progress, giving similar responses [40]. More recently, MWCNTs-7 were administered by a single intrascrotal injection in 7 Fischer 344 rats (240 μg/rat) maintained for an observation period of 52 weeks [16]. Dimensions were 82% of the MWCNTs with a diameter between 70–110 nm, and 72.5% between 1–4 μm in length. Five vehicle-treated controls and 7 UICC crocidolite-treated rats (470 μg/rat) were also studied. The overall incidence of mesotheliomas was 86% in MWCNT-treated rats while no mesothelioma was found in vehicle- or crocidolite-treated rats. This method of exposure of mesothelial cells is not usually used to assess a carcinogenic potency of fibres. However, injury at the scrotal mesothelium is used as a method to investigate the repair mechanism of peritoneal mesothelium [42,43]. Further data are clearly needed to improve our knowledge of the effects of these MWCNTs on mesothelial cells *in vivo*.

##### Effects on mesothelial cells *in vitro*

To the best of our knowledge, four studies have reported *in vitro *effects on mesothelial cells. DNA breakage and DNA repair were found in both human normal and malignant mesothelial cells exposed to SWCNTs, as well as cell activation *via *AP-1, NF-κB and Akt [44]. Another study concluded that there was alteration of cell viability and decreased cell proliferation in human mesothelioma cells exposed to SWCNTs [45]. Three studies reported cytotoxicity on human normal mesothelial cells, malignant mesothelioma cell line, and on largeTSV40-transformed mesothelial cells (Met-5A) [44,46,47]. It is noteworthy that the same raw CNT material with different degrees of dispersion exerted different cytotoxicity on a human mesothelioma cell line [47]. In this study, the toxicity of CNT-bundles (well-dispersed material with a bundle diameter of around 20 nm) was less than that of CNT-agglomerates (densely roped aggregates with a rope diameter in the micron-range). CNTs appear to be taken up by different cell types and diverse *in vitro *effects have been associated with CNTs uptake [2,45]. However, the cellular uptake of CNTs is controversial. Both absence and significant uptake have been reported, as recently discussed [1]. Uptake is likely dependent on interactions between cellular receptors and cell surface functions, and CNTs surface reactivity. A variety of cell surface functions may be found, depending on the cell type. CNTs may also carry diverse reactive groups. Different sorts of chemicals and biological molecules are currently used to disperse CNTs that may modify the CNTs surface. Hence cell-CNT interactions are dependent on a number of intrinsic and extrinsic parameters. It should be recalled that modification of the surface of asbestos fibres modulates the cell responses [48-52]. In macrophages, the scavenger receptor with collagenous structure (MARCO) seems to play an important role in pulmonary damage induced by inorganic particles [53] and may be involved in interaction between MWCNTs and plasma membrane of macrophages [54]. In mesothelial cells, integrin receptors were reported to interact with asbestos fibres [50,55]. Recently, no particle internalisation was evidenced in largeTSV40-transformed mesothelial cells (MeT-5A) exposed to MWCNTs, despite cytotoxicity [46]. Further studies are necessary to clarify these controversial results, as fibre internalisation is an important process accounting for the adverse cellular effects of particles, and more data are needed to determine the interactions of CNT with mesothelial cells.

#### Biological effects in other systems

##### Inflammation

Several studies have investigated the inflammatory response provoked by CNT exposure, conducted on mice or rats exposed *via *intratracheal instillation or inhalation. Several reviews may be consulted for more details [1,2,4-8]. Some recent data are summarized in Table 1[30,56-59].

**Table 1:** Summary of recent *in vivo *experiments carried out with CNTs

Type of CNT	System	Summary results	Reference
SWCNTs (Carbon nanotech Inc. Tx). Ø: 0.8–1.2 nm L: 0.1–1 μm	Pharyngeal deposition in C57Bl/6 mice lung (40 μg/mouse). Observation 4 hours post exposure.	Gene expression in lung and blood: Upregulation of genes involved in inflammation, oxidative stress, coagulation, tissue remodeling. Increased percentage of polymorphonuclear leucocytes (PMN) in blood and bronchoalveolar lavage (BAL).	[30]

SWCNTs. 240 nm (mode, aerodynamic diameter; in number)	Inhalation (4 days) in mice – 5 mg/m^3^ Short and mean term responses (1, 7, 28 days)	Lung analysis: Inflammation – Granulomas – Fibrosis – Mutation of K-*ras*	[58]
		
4.2 μm (mode, aerodynamic diameter; in mass)	Laryngeal deposition (10 μg/mouse). Short and mean term responses (1, 7, 28 days)	Lung analysis: Inflammation – Granulomas – Fibrosis - No mutation of K-*ras*. Lower effects compared to inhalation.	

MWCNTs. Ø: 40–60 nm L:0.5–500 μm	Intratracheal deposition in rats. One to 7 mg/kg. Short/mean term responses (1 to 90 days)	Inflammation; dose-dependent thickening of the alveolar lining Particles still present after 3 months	[56]

MWCNTs grinded, unheated, heated to 600°C, 2400°C; 2400°C then grinded. Ø: 20–50 nm L: 0.7 ± 0.07 μm	Intratracheal deposition in rats, 2 mg/rat. Short-term response (3 days); mean-term (60 days)	Inflammation (3 days). Granulomas (60 days). Effects of heated CNTs lower than unheated. Grinding restored the effects.	[57]

MWCNTs. Ø: 40–60 nm L:0.5–500 μm	Intratracheal deposition in rats. One to 7 mg/kg. Short/mean term responses (1 to 90 days)	Inflammation; dose-dependent thickening of the alveolar lining Particles still present after 3 months	[56]

MWCNTs (Mitsui & Co., LDT) Ø: ≅ 80 nm L: 10–20 μm	Pharyngeal deposition in C57Bl/6 mice lung (40 μg/mouse). Observation 4 hours post exposure.	Gene expression in lung and blood: Upregulation of genes involved in inflammation, oxidative stress, coagulation, tissue remodeling. Increased percentage of polymorphonuclear leucocytes (PMN) in blood and bronchoalveolar lavage (BAL).	[30]

MWCNTs Shenzhen nanotech Ø: 500 nm; L: 10 μm	Inhalation (≈ 32 mg/m^3^) in mice for 5, 10, 15 days; deposition ≈ 0.07, 0.14; 0.24 μg/mouse. Short-term response (8, 16, 24 days)	Small aggregates entering the alveolar wall Cell proliferation and thickening of alveolar walls	[59]
		
	Tracheal deposition: 50 μg/mouse	Eight and 16 days: clumps deposited on lining wall of bronchi, no inflammation – 24 days: inflammation. Clumps in the alveoli destruction of alveolar structure	

Regarding the large applications of CNTs and the known adverse effects of fine particulate matter, the potential effects of CNTs have also been investigated on systems other than respiratory [1,2]. A recent study suggests that deposition of both SWCNTs and MWCNTs produce a systemic response, which may affect the cardiovascular system [30].

##### Genotoxicity

Both *in vivo *and *in vitro *effects of CNTs suggest a possible genotoxic effect, related to inflammatory responses and production of reactive oxygen species, as persistent inflammation is considered to increase the carcinogenic risk [60].

*In vivo*, a mutation of the *K-ras *oncogene was observed in mice exposed to SWCNTs by inhalation, and chromosomal aberrations were detected in type II pneumocytes after intratracheal deposition of MWCNTs in mice [58,61]. Several *in vitro *studies have reported a genotoxic potency using different cell types (Table 2) [44,46,54,57,61-70]. Activation of DNA repair processes and mutagenesis of the adenine phosphoribosyl transferase gene was found in mouse embryonic stem cells [69]. Genotoxicity as assessed by the cytokinesis-block micronucleus test, was found in rat lung epithelial cells exposed to MWCNTs [57]. Micronuclei formation occurred in human epithelial cells (MCF-7) treated with MWCNTs, and a pancentrometric probe analysis demonstrated both chromosome breakage and chromosome loss [61]. DNA damage was also reported in SWCNT-treated mouse embryo fibroblasts and in CNT-treated bronchial epithelial BEAS 2B cells [63,67]. No mutation or DNA breakage was found in a FE1-Mutatrade markMouse lung epithelial cell line exposed to SWCNTs but purine oxidation was detected with the Comet assay [71].

**Table 2:** Summary of recent *in vitro *experiments carried out with CNTs

Type of CNT	System	Summary results	Reference
SWCNTs (HiPco), (CNI Inc.). Ø: 0.4–1.2 nm L: 1–3 μm	Lung hamster fibroblasts (V79)	Cytotoxicity (time and dose dependent) DNA breakage (comet assay) No significant enhancement of micronuclei	[62]

SWCNTs (50% SWCNT, about 40% other nanotubes). Ø: 1.1 nm, L: 0.5–100 μm	BEAS 2B human bronchial epithelial cells	Dose-dependent decrease in cell viability. Dose-dependent DNA damage. No formation of micronuclei	[63]

SWCNTs (NIST) Ø: 1.4 nm, L:2–5 μm	Normal human mesothelial cells and human mesothelioma cell line	Cell death. DNA lesions Stress response activation	[44]

SWCNTs. Folate conjugated. Ø: 1–3 nm, L: 100 – 200 nm	HepG2 cells (express folate receptor)	No toxicity if < 50 μg/ml. Dose-dependent apoptosis. Kinetics of SWCNT internalisation: Mb → cytoplasm → extracellular	[64]

SWCNTs (HiPco)	Human lung epithelial cells A549 and immortalised NHBE	Decreased inflammatory response in TNF alpha-stimulated cells	[65]

SWCNTs Mitsui & Co., Ltd Size unspecified	Human aortic endothelial cells	Internalisation: CNTs identified in the cytoplasm. Cytotoxicity. IL-8 release. Actin filament and Ecadherin disruption. Reduced tubule formation.	[66]

SWCNTs	Mouse embryo fibroblasts	Low cytotoxicity. DNA damage (comet assay) Oxidative stress	[67]

MWCNTs. Ø: 67 nm	Mouse macrophages (J774.1).	No MAPKs activation; no apoptosis. Interaction with membrane receptors (MARCO) and plasma membrane destruction	[54]

MWCNTs. Ø:11.3 nm L:0.7 μm	Human epithelial cells (MCF-7)	Chromosomal aberrations (micronuclei) showing chromosome breakage and loss of whole chromosomes	[61]

MWCNTs (C100, Arkema). Ø: 12 nm, L: 0.1–13 μm	Human epithelial (A549) and Large T SV40 transformed mesothelial (Met-5A) cells	Decrease in cell viability (mitochondrial alteration) without apoptosis. No oxidative stress. No MWCNT internalisation	[46]

MWCNTs grinded, unheated, heated to 600°C, 2400°C; 2400°C then grinded. Ø: 20–50 nm; L: 0.7 ± 0.07 μm	Rat lung epithelial cells.	Chromosomal aberrations (micronuclei) Lower effects with 2400°C sample in comparison to 600°C and unheated	[57]

MWCNTs. Ø: 100–200 nm, L:a few μm	Human epithelial cells (A549)	DNA breakage (comets). No oxidative DNA lesions	[68]

MWCNTs (Tsinghua & Nananfeng, Cine)	Mouse embryonic cells (ES)	P53 activation. Induction of DNA repair. Mutations (adenine phosphoribosyl transferase)	[69]

MWCNTs Mitsui & Co., Ltd Size unspecified	Human aortic endothelial cells	Cytotoxicity. IL-8 release. Actin filament and Ecadherin disruption. Reduced tubule formation.	[66]

MWCNTs	Human pneumocytes A549	Decrease in cell viability Internalisation	[70]

Investigations of the mutagenic potency of MWCNTs using bacterial test systems did not reveal mutagenic activity [72,73]. These bacterial assays may not be fully relevant to evaluate genotoxicity of particles. Previous results with bacterial cells were generally not or only moderately positive with asbestos fibres [74].

## Asbestos fibres' mechanism of action

### The asbestos legacy

Numerous publications on the mechanism of action of asbestos fibres have emphasized several responses associated with the mechanism of toxicity at the serosal level. They make it clear that two aspects must be considered: the biological response and the particle status. The first depends on several factors that include the fate of asbestos fibres following inhalation, i.e., their ability to reach the pleura. It is well know that deposition, clearance and translocation of fibres are dependent on biological mechanisms and partners (mucociliary transport, phagocytic cells), but also on fibre parameters, especially fibre dimensions. Short fibres are more easily internalised by macrophages than long fibres, and long fibres possibly involve "frustrated phagocytosis." The biopersistence of fibres is linked to both their dimensions and stability in the biological milieu.

### Fate of asbestos fibres

Regarding industrial uses and commercial applications of asbestos fibres, the main risk of contamination is linked to the inhalation route. In general, particle deposition depends on aerodynamic considerations. Several authors have studied the mechanism of fibre deposition and retention in the lungs [75-78]. Once deposited in the lung, asbestos fibres may be translocated into different organs and tissues, including the pleura. This was demonstrated in animals following inhalation or intratracheal deposition [79], and in humans by investigation of fibre retention in different body compartments including the pleura [80-82]. A recent paper discusses the translocation pathways of asbestos fibres to the pleura [83]. Translocation appears to be due to trans-cell migration and lymphatic circulation. These authors propose that fibres deposited in the alveolar space can be translocated to the interstitium, down the gradient of physiological water absorption. This transfer is facilitated when the epithelial layer is damaged. Once in the interstitium, fibres can be distributed to different organs. Fibres can be cleared from the interstitium *via *the lymphatic system and enter the capillaries as inflammation increases the interstitial pressure, allowing the fibres to migrate and be distributed throughout the whole body. Therefore, fibres can reach the pleura *via *the capillary system and transfer through the visceral pleura. The parietal pleural has pores of relatively large diameter (about 150–200 nm), and the pleural fluid drainage goes through stomatas where particles are found to be concentrated.

Translocation of CNTs to the pleura can be assumed, as asbestos fibres are not the only particles to be translocated to this site. Migration was observed after inhalation of refractory ceramic fibres and NMVF10a fibreglass in hamsters and rats, and anthracotic areas ("black spots") containing particulate matter are present in human pleura [81,84-87]. One important point for the study of CNT toxicity is therefore to determine their ability to be distributed in the body and to reach the pleura. It is likely that the CNT aggregation state will modulate the rate of translocation. Recent experiments comparing inhalation and tracheal or pharyngeal deposition of CNTs concluded that the different effects were likely related to a difference in the dispersion and aggregation state of the CNTs [58,59]. It can also be assumed that the CNT pre-treatments used for particle dispersion will also influence the biodistribution of these particles. Moreover, it must be kept in mind that CNT exposure takes place *via *routes other than inhalation, which ought to be investigated.

### Biological and genomic effects of asbestos fibres on mesothelial cells

#### Inflammation and mesothelial cell activation

Many authors have described the inflammatory processes occurring in the lung and in the pleura, and shown that fibres can interact with mesothelial cells in culture conditions. Fibre deposition in the lung is followed by the recruitment of inflammatory cells, which produce several factors: ROS (reactive oxygen species), RNS (reactive nitrogen species), clastogenic factors and cytokines that may stimulate and/or damage neighbouring mesothelial cells. Fibres also may produce ROS. Moreover, mesothelial cells respond by fibre internalisation according to a phagocytic process associated with oxidative reactions [34,88-93].

In this situation, mesothelial cells adapt to the oxidative environment by oxidative stress, increasing oxidant defences and decreasing natural ROS and RNS production. At the same time, several regulatory pathways are activated: signalling pathways (MAPKs) associated with cell proliferation and apoptosis, and DNA repair and control of cell cycle progression in response to DNA damage [94,95]. These different reactions are the consequence of 2 types of interactions: between cells (inflammatory cells/mesothelial cells) and between cells and fibres. As neoplastic transformation is linked to genetic damage and requires proliferation steps, comparison between the genotoxic effects of asbestos and CNTs might provide clues making it possible to develop hypotheses about the potential effects of CNTs.

#### Genotoxicity

Many investigations have focused on DNA damage provoked by asbestos fibres in mesothelial cells. Several studies have demonstrated different types of DNA damage (DNA breakage, base oxidation), and perturbation of the mitotic process [94,95], showing that base oxidation and DNA breakage (single strand and double strand breaks) were detected in asbestos-treated mesothelial cells [95-100]. These may be due to ROS/RNS production and to the mesothelial cells' ability to phagocytise asbestos fibres. Fibre uptake does not abolish the mitotic process as some fibres are found in dividing mesothelial cells. Moreover, extensive chromosome damage was described. A list of chromosome abnormalities has been reported by different authors. Asbestos fibres produce structural chromosome alterations; significant enhancement of aneuploid cells, abnormal anaphases and telophases [101-105]. Induction of micronuclei by all types of asbestos in primary cultures of human mesothelial cells has been reported by Poser *et al*. [106]. Other studies have shown genomic alterations in asbestos-treated human mesothelial cells. Loss of heterozygosity was detected as asbestos-induced mutations in a human mesothelioma cell line [107]. Using 3D reconstruction, Cortez *et al*. recently reported mitotic abnormalities, centrosome amplification and aneuploid cell formation in lung carcinoma cells, even with long periods of recovery post-treatment [108]. These findings are similar to earlier reports concerning rat pleural mesothelial cells and using less powerful methods.

#### Gene expression in asbestos-treated mesothelial cells

A few studies have investigated gene expression in asbestos-treated mesothelial cells using microarray analysis (Table 3) [109-111]. They confirmed results obtained in studies focusing on given types of damages. Modulation of several biological processes were observed. They were associated with inflammatory, proliferation, DNA repair and cell adhesion pathways. Further studies comparing the cell response to CNTs and to the different types of asbestos fibres are likely to be informative in order to approach the possible effects of CNTs.

**Table 3:** Summary of *in vitro *experiments related to gene expression in crocidolite-treated mesothelial cells

System	Summary results	Reference
Human mesothelial cells (LP9/TERT-1) exposed to low and high concentrations (15 and 75 μm^2^/cm^2 ^per dish) for 8 or 24 h Oligonucleotide microarray analysis	ATF3-dependent modulation of inflammatory cytokines and growth factor production	[109]

Human SV40-immortalized pleural mesothelial (MeT-5A) cells exposed to 1 μg/cm^2 ^dish for 1–48 h Oligonucleotide microarray analysis	1 h: upregulation of nucleosome assembly, translational initiation, transcription, I-kappaB kinase/NF-kappaB cascade, survival 48 h: downregulation of cytoskeletal anchoring, transcription, survival	[110]

Normal rat pleural mesothelial cells exposed to 5 μg/cm^2 ^dish for 24 h Oligonucleotide microarray analysis	Induction of fra-1-linked cd44 and c-met expression	[111]

#### Gene alterations in mesothelioma

Epidemiological studies have shown that MM is a consequence of asbestos exposure in a majority of cases [18-24]. This led us to assume that genomic alterations found in MM could be linked to the effect of asbestos fibres. The identification of these changes can provide insight into the molecular mechanism of action of asbestos on mesothelial cells. MM cells exhibit frequent alterations in tumour suppressor genes found at the *INK4 *locus, and often the type of alteration is deletions. *NF2 *is another frequently inactivated tumour suppressor gene in MM cells. Germinal mutations in *NF2 *are responsible for type 2 neurofibromatosis, but NF2 patients are not prone to develop mesothelioma. *TP53 *is mutated less often in MM cells.

To investigate whether genetic alterations in mesothelioma might be relevant to the effect of asbestos fibres, animal models of human MM are developed. Mesotheliomas develop following exposure, by intraperitoneal injection, of hemizygous *NF2 *mice to asbestos fibres [112,113]. This made it possible to compare characteristics of mouse and human mesotheliomas.

Histologically, very similar tumours were observed, and the genomic alterations in the tumour suppressor genes investigated were very close to those observed in human MM. These gene are involved in the control of cell cycle and junction stability. Regarding the function of the gene, it might be of interest to determine the consequences of CNT exposure on cell cycle progression and cell architecture.

## Asbestos fibre characteristics related to disease

If one looks at fibre parameters, several features appear to be shared by CNTs and asbestos fibres. To compare CNTs and asbestos fibres in relation to toxic potential, we should focus on the asbestos characteristics modulating asbestos toxicity. Shape, size, chemistry and surface reactivity are all related to cell and tissue responses to asbestos fibres.

### Shape

CNTs have a thin and elongated shape compatible with a fibre, according to the WHO fibre definition of a particle with parallel edges and an aspect ratio (length/diameter) greater than three. It seems that CNTs are prone to form aggregates, ropes and clumps, a feature that is not fully similar to asbestos, which forms bundles of rather well-organized structures. The length of CNTs may vary, reaching up to several micrometers or longer [7,114]. Accordingly, "frustrated phagocytosis" was observed in cells engulfing long CNTs [37].

### Size

The diameters of asbestos fibres fall in the nanosize range. If one refers to the dimensions of the UICC samples, which have been used in a number of animal and cell studies, the diameter of chrysotile fibres was less than about 100 nm, and 200 nm for crocidolite. Length depended on the sample, but generally averaged several micrometers. However, there was a significant range in length and a small percentage of fibres longer than 10 μm were generally present.

### Chemistry

Metals are considered to be important elements to account for fibre toxicity. Iron content, either structural or as contaminant, may be linked to the formation of ROS and RNS. Depending on the method of production, CNTs may contain metals as contaminants; moreover, they can be functionalized to acquire specific properties. Data in the literature show a wide qualitative and quantitative diversity of metal contaminants in the chemical composition of CNT samples, emphasizing the importance of using well-defined samples for toxicological analyses [7].

### Surface reactivity

Surface reactivity is an important parameter in asbestos-related effects. The production of ROS and RNS was mentioned above. It is interesting to note that some studies indicate that, in contrast to asbestos, CNTs quench ROS in an acellular system generating hydroxyl radicals [115]. While asbestos fibres are hydrophilic, CNTs, unless functionalized, are hydrophobic. As a result, CNTs are often treated with dispersing agents prior to exposing cells or animals to CNTs suspended in aqueous medium.

Asbestos fibres' ability to adsorb biological molecules is another fibre parameter to take into consideration. Asbestos adsorbs proteins and phospholipids, which has consequences on cell-fibre interactions. An enhancement of biological effects can be observed (particle internalisation, cytotoxicity), as well as a reduction of toxicity [116-119].

Asbestos bodies are structures found in the lung of asbestos-exposed subjects. They consist of an asbestos fibre core surrounded by a complex coat produced by the cell and tissue reaction; they are made of apatite mineralization and protein aggregation (hemosiderin, ferritin). These structures are more likely formed on amphiboles rather than on chrysotile. They are not specific to asbestos, as they have been reported in other fibrous and non-fibrous particles. It would be of interest to know whether these structures could be formed on CNTs [120-122].

### Biopersistence

While biopersistence is not an intrinsic particle parameter, it has received attention for the evaluation of the carcinogenic potency of manmade vitreous fibres (MMVFs) [123,124]. Biopersistence in the lung is the result of a clearance mechanism and the behaviour of fibres in the biological medium. Clearance depends on particle uptake by scavenger cells; it is then modulated by the fibre size and toxicity (short particles are eliminated following uptake by macrophages; cytotoxic particles impair the process). The behaviour of the fibres is also size-dependent (fibre dimensions govern the mechanism and site of deposition in the lung), as well as dependent on the fibre structure and chemistry (these parameters modulate the stability of the particles in the biological medium). Some chemical elements may dissolve and reduce fibre strength, breaking the fibres into smaller fragments. Finally, biopersistence modulates the amount of fibre retained in the lung and the time it remains in the lung. To date, CNTs have been considered biopersistent, but further studies are needed to determine the relevance of this parameter in the context of human exposure to CNTs [57].

## Discussion

CNTs are valuable industrial products with multiple applications in the field of nanotechnologies, yet legitimate concerns about their potential adverse effects on human health need to be addressed. The risk of MM, a primary pleural carcinoma linked to asbestos exposure, must be examined in light of the physical nature of CNTs, which are elongated and ultrafine, and the fact that human beings may be exposed to CNTs through inhalation. While not yet definitive, data are now available providing information on the pulmonary and cellular effects of CNTs, which may be compared to those of asbestos fibres. Moreover, the asbestos fibre characteristics involved in the toxic processes may be compared to those of CNTs to determine their similarities. These comparisons make it possible to develop hypotheses about common and different mechanisms of action. A summary of comparisons between CNTs and asbestos is provided in Tables 4 and 5.

**Table 4:** Comparison between physical and chemical parameters of asbestos and CNTs

***Parameter***	***Comparison***
Shape	Both are elongated particles; fibre shaped.

Dimensions	Asbestos fibre diameter: range of 100 nm. Chrysotile fibrils: ≅ 50 nm of diameter. Same order as MWCNTs.

Structure	Chrysotile: multi-layered rolled sheets of brucite (MgOH_2_) and silicon oxide (SiO_2_). Important aggregation with CNTs, which may form more entangled bundles, ropes, than asbestos.

Chemistry	Different chemistry. Possibility of metal impurities in both asbestos and CNTs.

Surface reactivity	Both show sorptive properties to biological molecules. ROS production: no definitive answer for CNTs.

**Table 5:** Comparison between biological effects of asbestos and CNTs

***Cell/tissue response***	***Comparison***
Particle uptake	Demonstrated with both types. Conflicting results with CNTs. Exocytosis found with CNTs, so far not investigated with asbestos.

Cytotoxicity	Both cytotoxic.

DNA damage, mutation, gene interaction	Found with both asbestos and CNTs.

Transfection	Gene transfer is with asbestos. CNT gene knockdown.

Biodistribution	Both types are cleared *via *the lymphatic system and found in different organs

Inflammation, granulomas, fibrosis	Found with both asbestos and CNTs. Both types show dependence of biological effects with fibre dimensions: bioactivity of long fibres.

Cancer	MM found with both asbestos and CNTs by peritoneal exposure.

A paradigm for the health effects of HARN has emerged from toxicology studies of industrial fibres, including asbestos. A recent report reviewed state-of-the-art knowledge of the toxicity of asbestos and HARN [3]. This clearly suggest a community of toxicological features and concern between HARN of different origin and composition. The reader will find in this quoted review additional information on other HARN (nanowires, nanorods) and the proposal for a research strategy to determine the potential toxicity of HARN.

### Shape, structure and chemistry

Both CNTs and asbestos particles share fibrous morphology, and their dimensions are in the same range. CNTs are manufactured in two main forms, SWCNTs and MWCNTs. A SWCNT is a single-layer graphene sheet rolled up in a cylindrical shape, whereas a MWCNT contains several layers [125]. The structure of chrysotile presents similarities with MWCNT. CNT samples may have much higher length than asbestos fibrils and form clumps resulting in different presentation and tissue penetration. One role of CNT sample dispersion to modulate biological effects is suggested by the results reported from *in vivo *experiments studying inhalation and intratracheal deposition.

### Biodistribution

Similarly to asbestos fibres, CNTs may be deposited and retained in the lung after inhalation. So far, there is no definitive data on their migration and long-term retention, and on their translocation to the pleura. Interaction with mesothelial cells is likely important to account for asbestos pathogenicity; however, distant effects after deposition in the lung have been reported. As already mentioned, CNTs are the subject of scientific interest for a large number of already mature or potential applications. One paradox is that biological studies with CNTs are designed to investigate both adverse (exposure to toxic dust) and beneficial (nanomedicine) effects. These different types of studies show that MWCNTs are concentrated in the lymph nodes after deposition in the lung, and that functionalized MWCNTs also accumulate in lymph nodes after subcutaneous injection [29,126]. CNT biodistribution has been studied following intraperitoneal or intravenous injection in mice. CNTs are distributed throughout the entire body and cleared *via *urine excretion. McDevitt *et al*. found an accumulation of labelled SWCNTs in the kidney, liver, spleen and, to a lesser extent, in bone [127]. There is to date no reason to exclude the possibility of CNT translocation to the serosa.

In a recent paper, Hankin *et al*. [128] summarized the research required into the mechanism of translocation of nanoparticles across the respiratory epithelium, and the resulting possible effects in and beyond the lung. The authors provide recommendations to develop research on translocation and penetration of nanoparticles that take into consideration the parameters allowing a robust interpretation of the data.

### The relationship between structure and biological effects

Based on our present knowledge, a comparison between cell responses to SWCNTs and MWCNTs cannot be established. This is partly due to the limited number of investigations carried out with both types of nanotubes in the same assays. Nevertheless, both types are able to induce biological responses in one or several cell types, and in the lung. While studying the biodistribution of MWCNTs following intraperitoneal injection, Guo *et al*. compared different results obtained with both SWCNTs and MWCNTs [129]. These authors suggest that toxic responses observed in the kidney in some studies may depend on whether CNTs are functionalized, a procedure that may improve the biocompatibility.

Surface functionalization, purity and treatment of CNTs appear to modulate the biological response, as found in different studies [115,130,131]. The surface modifications of CNTs developed in the field of nanomedicine studies are of interest to learn about interactions between CNTs and cells or organelles. It is already known that surface changes influences cell responses. Viability of neuroblastoma cells was not affected by pure MWCNTs (99% purity). Viability and proliferation were reduced after acid treatment or when MWCNTs of lower purity were used (97%) [131]. Acute pulmonary toxicity and genotoxicity of MWCNTs were reduced upon heating but restored upon grinding, in relation with surface defects[57]

Studies carried out with asbestos have demonstrated that long and thin fibres are more toxic than short fibres, without excluding potential toxicity for short fibres. Limits of 4 μm or 8 μm in length have been proposed, mainly based on *in vivo *experiments. CNTs can fulfil these length criteria, and similarly to asbestos, long CNTs were more active than short CNTs [37]. More data on size-dependent biological effects of CNTs will be of great interest.

Surface reactivity of asbestos fibres has been largely advanced as a key parameter accounting for their toxicity in terms of ROS production and sorptive abilities. ROS production is associated with cytotoxicity, cell activation, and chromosome and DNA damage. Conflicting data are found with CNTs, as both production and scavenging of ROS were described. CNTs are a large family regarding their method of generation, treatment and functionalization. Hence the surface reactivity of CNTs towards biological systems will be largely dependent on the type of nanotubes. This may be maximized by treatments to disperse CNTs prior to use for biological studies. Different CNTs samples may have more heterogeneous surface activities than asbestos.

### Biological effects

Available data in the literature concerning the effects of CNTs on mesothelial cells remain limited. Several effects of asbestos fibres, especially genetic damage, are related to fibre internalisation. While asbestos fibres are clearly internalized by mesothelial cells, there is no definitive data on CNT uptake by these cells. The physico-chemical properties of CNTs are likely to take into consideration in the occurrence of this physiological process.

Animals and cell systems exposed to CNTs appear to develop several responses similar to those observed with asbestos. There is some evidence that CNTs produce inflammation and mesothelioma when inoculated in the peritoneal cavity of mice. This result is consistent with inflammatory potency after inhalation or intratracheal instillation. Several CNT-exposed mammalian cells respond in culture conditions by inflammatory reactions and oxidative stress. Genotoxic potency has been reported in different cell types, including mesothelial cells, as well as in studies conducted *in vivo*. Hence CNTs used in experimental systems fulfil several criteria to elicit tissue injury, including the mesothelium.

## Conclusion

The link between asbestos effects and mesothelioma has been attributed to several mechanisms. This link has been investigated using different animal models and cell types, and substantial similarities exist in the responses of the different cell types. Several fibre properties have been linked to adverse effects. Shape, dimension and surface reactivity are all important parameters. Reactive species may produce DNA lesions (base oxidation, breaks); their origin is related both to fibre surface reactivity and phagocytosis. Inflammation contributes to the production of ROS/RNS. Chromosome damage appears to be of major importance to account for the significant effects of asbestos. Gene deletions and recombinations might result from these effects. Integrity of some cell processes may be critical in the response to asbestos: dynamics of the cell membrane (fibre uptake, cell division) and control of cell division (check points, chromosome segregation processes, repair of DNA breakage, cytokinesis). In view of findings of a much less hazard of short than some long/non-biopersistent HARN, more information is needed on the CNTs' characteristics and conditions to which we may be exposed. Based on available data in the literature and knowledge of the mechanisms of action of asbestos fibres, it appears that CNTs may elicit responses that are similar to those caused by asbestos fibres.

In this review, for the sake of brevity, CNTs have been considered as a single entity. However, there is a large degree of heterogeneity within the CNT family. The legacy of our knowledge on the mechanism of action of asbestos prompts us to recognize some similarities between these two types of particles. Nevertheless, in view of the diverse uses of CNTs, toxicological studies should be carried out in the context of the respective applications, taking into consideration the possible interactions between the target tissue and the nanotubes, and their possible biodistribution. An evaluation of the conditions and type of exposure to CNTs thus appears mandatory to focus clearly on the safety and health issues. Exposure by inhalation for aerosolized CNTs must take into consideration both lung and pleural diseases.

## Competing interests

The authors declare that they have no competing interests.

## Authors' contributions

MCJ contributed to the design and drafting of the manuscript. AR participated in the acquisition of data on asbestos on mesothelial cells and the genetics of mesothelioma cells. JD analysed the data in the literature on gene expression.
